# Anti-Aging Potential of Biogenic Selenium Nanoparticles and Selenium/Polysaccharides Nanoconjugate Biosynthesized by *Bacillus subtilis* Isolated from Selenium-Rich Soil

**DOI:** 10.3390/antiox15070866

**Published:** 2026-07-11

**Authors:** Yiming Luo, Chengbin Feng, Mengze Liu, Pengfei Zhao, Danfei Huang

**Affiliations:** 1 China-Canada Joint Lab of Food Science and Technology (Nanchang), Nanchang University, 235 Nanjing East Road, Nanchang 330047, China; qw12345q2@163.com (Y.L.); fengchengbin@email.ncu.edu.cn (C.F.); liumemgzencu@163.com (M.L.); zhaopf2026@126.com (P.Z.); 2 State Key Laboratory of Food Science and Resources, International Institute of Food Innovation Co., Ltd., 235 Nanjing East Road, Nanchang 330047, China

**Keywords:** selenium nanoparticles, biosynthesis, *Bacillus subtilis*, antioxidant, D-galactose

## Abstract

Selenium nanoparticles (SeNPs), which are nanoscale particles of elemental selenium (Se^0^), combine the biological activity of selenium with the distinctive properties inherent in nanomaterials, thereby making SeNPs a more promising candidate for advancing selenium-based resources. In this work, several strains of *Bacillus* with sodium selenite-reducing ability were isolated from selenium-rich soils. *Bacillus subtilis* ESNS-2 with high selenium tolerance was selected as the target strain. Under culture conditions supplemented with 5 mmol/L sodium selenite, this strain exhibited a reduction efficiency of 75.4 ± 0.6% over 24 h. The produced bioSeNPs were purified, decorated using polysaccharides from the seeds of *Plantago asiatica* L. (PLP), and subsequently systematically characterized using various means. The results revealed that the prepared PLP-bioSeNPs were regularly spherical elemental selenium particles, with an average particle size of 96.9 ± 1.1 nm, a PDI of 0.108 ± 0.003, and a zeta potential of −19.7 ± 0.4 mV. Characterization confirmed that they exhibited excellent dispersibility and stability. In vitro antioxidant assays, both bioSeNPs and the PLP-bioSeNP complex demonstrated pronounced dose-dependent scavenging activity against DPPH• and ABTS^+^• radicals. In a D-galactose-induced aging mouse model, both bioSeNPs and PLP-bioSeNPs alleviated D-galactose-induced hepatic and cerebral damage, as well as associated behavioral deficits, through the modulation of oxidative stress balance and suppression of inflammation. This study successfully accomplished the efficient production of SeNPs utilizing *Bacillus subtilis* ESNS-2. The modification of PLP provided innovative strategies for the development of macromolecular drugs, with a specific emphasis on enhancing stability.

## 1. Introduction

Selenium constitutes an indispensable trace element for human health, which is incorporated into selenoproteins and functions as an essential cofactor for fundamental enzymatic reactions in vivo [1]. Beyond its robust antioxidant capacity, selenium demonstrates a spectrum of significant biological activities such as anticancer activity, immunomodulatory function, enhancement of reproductive performance, and cardioprotective property [2,3]. Given that the nutritional status of selenium in the human body is predominantly determined by dietary intake, selenium-enriched foods have garnered increasing prominence in recent years [4]. The chemical speciation of selenium, which includes inorganic selenium (e.g., sodium selenite and sodium selenate), elemental selenium (Se^0^), and organic selenium (e.g., selenomethionine and selenocysteine), determines its absorption, metabolic efficiency, and biological effects [5,6,7]. Unlike the pronounced toxicity of inorganic selenium [8] and the instability of organic selenium [9], selenium nanoparticles (SeNPs), which are nanoscale particles of elemental selenium (Se^0^) that generally range in diameter from 1 to 100 nm, have garnered considerable attention recently owing to their high biocompatibility, modifiable surface, and lower toxicity than inorganic and organic selenium at equimolar doses [10,11].

At present, various methodologies including physical, chemical and biological methods have been developed for the fabrication of SeNPs, among which physical and chemical synthesis techniques exhibit apparent disadvantages, such as elevated production costs, adverse environmental impacts, substantial energy consumption, poor stability and compromised biocompatibility [12,13,14]. Recently, the biosynthesis of SeNPs utilizing living organisms, including plants, algae [15], fungi [16], bacteria, and actinomycetes [17], has garnered increasing attention owing to its advantages in terms of affordability, accessibility, environmental safety, and low toxicity. Most selenium-tolerant microorganisms initially detoxify selenite into less toxic organic selenium within cells. Nevertheless, upon exceeding their selenium tolerance threshold, these microorganisms initiate the synthesis of SeNPs, either intracellularly or extracellularly, which demonstrates a propensity for aggregation [18]. The reduction process entails the enzymatic conversion of selenite (Se^4+^) to elemental selenium (Se^0^), proceeding through the intermediate formation of selenide (Se^2−^). Additionally, it is also hypothesized that microorganisms synthesized compounds, such as organic acids, polysaccharides [19] and proteins [20], participate in the reduction process and function as stabilizing agents.

Among microbial producers of SeNPs, *Bacillus* sp. have garnered considerable interest owing to their notable tolerance for selenium and efficient biosynthesis capabilities [21,22]. However, some bacteria within the *Bacillus* sp. such as *Bacillus* cereus [23] have been identified as posing potential safety risks that limit their application in the field of human health. Consequently, the development of a safe and efficacious methodology for employing *Bacillus* sp. in the synthesis of SeNPs holds considerable promise for advancing human health and nutrition.

*Plantago asiatica* L. is a herbaceous species with a cosmopolitan distribution. The seeds of *Plantago asiatica* L., which are employed for both medicinal and culinary purposes, demonstrate a spectrum of pharmacological characteristics [24], such as laxative, hypoglycemic, hypolipidemic, anti-inflammatory, immunomodulatory [25], and anti-tumor activities [26]. Polysaccharide extracted from the seeds of *P. asiatica* L. (PLP) is a neutral heteropolysaccharide that is rich in glycosidic bonds, monosaccharide residues, and linkage positions [27]. Consequently, PLP could serve as an optimal stabilizing agent for the encapsulation of bioSeNPs, thereby enhancing their stability and biological efficacy, and broadening their applicability in food supplements or additives.

Consequently, this study aimed to screen an efficient transformation strain from selenium-rich soils that is capable of reducing sodium selenite to SeNPs. Subsequently, the intracellular bioSeNPs were isolated, decorated with PLP, and their stability, antioxidant, and anti-aging properties were assessed.

## 2. Materials and Methods

### 2.1. Materials and Chemicals

Polysaccharide from the seeds of *Plantago asiatica* L. (PLP) was prepared following the methods described in previous reports by our research group [28]. It was characterized as a neutral heterogeneous polysaccharide with a complex structure and a protein content of 1.33 ± 0.23%. Sodium selenite, hydrochloric acid, sodium hydroxide, sodium chloride, ascorbic acid, physiological saline, 1,1-diphenyl-2-picrylhydrazyl (DPPH), and 2,2′-azinobis-(3-ethyl-benzothiazolin-6-sulfonic acid) diammonium salt (ABTS) were purchased from Aladdin Industrial Co., Ltd. (Shanghai, China). Polymyxin B, 50% yolk emulsion, Luria–Bertani (LB) broth culture medium, LB solid culture medium, and MYP (mannitol–yolk–polymyxin agar) culture medium were obtained from Qingdao Haibo Biotechnology Co., Ltd. (Qingdao, China). D-galactose was purchased from Sinopharm Chemical Reagents Co., Ltd. (Shanghai, China). A lipid oxidation (MDA) detection kit was provided by Byuntian Biotechnology Co., Ltd. (Wuhan, China). The superoxide dismutase (SOD) activity assay kit and glutathione peroxidase (GSH-Px) measurement kit were supplied by Nanjing Jiancheng Biological Company (Nanjing, China). The mouse lipopolysaccharide (LPS) assay kit and ELISA kits for interleukin-1β (IL-1β), interleukin-6 (IL-6), and tumor necrosis factor-alpha (TNF-α) were all obtained from Bioswamp Wuhan Bienle Biotechnology Co., Ltd. (Wuhan, China).

### 2.2. Bacterial Isolates and Growth Conditions

#### 2.2.1. Isolation of *Bacillus subtilis* from Selenium-Rich Soil

Soil specimens from selenium-rich areas in Enshi (Hubei province, average soil selenium content: 0.81 mg/kg [29]) and a selenium-rich industrial park in Ganzhou (Jiangxi province, average soil selenium content: 0.44 mg/kg [30]) were collected, then diluted with ultra-pure water and added to LB broth medium. After incubation at 37 °C with shaking for 18–24 h, the culture was heat-treated at 75–80 °C for 10–15 min. A total of 0.1 mL of the bacterial suspension was spread on LB plates and incubated at 37 °C for 24 h. Well-grown colonies were picked and purified via quadruple streaking. Colonies with irregular edges, gray-white color, rough surfaces, and dry type were examined microscopically and inoculated into MYP medium. Colonies showing rod-shaped cells with rounded ends, containing spores under microscopy, and appearing yellow with no precipitation ring on MYP medium were identified as *Bacillus subtilis* [31,32].

#### 2.2.2. Biosynthesis of SeNPs

Four *Bacillus subtilis* exhibiting robust growth and distinct red pigmentation were selected for SeNP synthesis capacity. After inoculation onto LB solid plates containing 5 mM sodium selenite (Na_2_SeO_3_) and incubation at 37 °C for 24 h, a single colony was inoculated into 5 mL LB liquid medium and incubated for 8 h at 37 °C in a rotary shaker (200 rpm). The strain was confirmed as *B. subtilis* through physiological and biochemical analysis [33] as well as 16S rRNA identification.

The strain named *Bacillus subtilis* ESNS-2 was transferred into 50 mL of LB broth (2% inoculum) supplemented with sodium selenite at a final concentration of 0, 20, 40, 60, 80 and 120 mM, and the cultures were incubated at 37 °C for 24 h with shaking at 200 rpm. The generation of elemental selenium was indicated by the medium turning reddish. The absorbance value of the fermentation broth at a 600 nm wavelength (OD value) was measured to determine the strain’s tolerance to sodium selenite. An OD_600nm_ value of <0.1 was used to indicate that the strain could not grow at that concentration.

### 2.3. Preparation of Selenium/Polysaccharides Nanoconjugate

#### 2.3.1. Purification of Biogenic Nano-Selenium (bioSeNPs)

After incubating *B. subtilis* ESNS-2 with 50 mL of LB broth (2% inoculum) supplemented with 5 mM sodium selenite at 37 °C for 48 h in a rotary shaker (100 rpm), the reaction mixture was centrifuged at 4500× *g* for 20 min and the pellets were collected. The pellets were rinsed three times with 1 × PBS buffer and then two times with 0.9% NaCl solution to get rid of the remaining supernatant. A total of 1 M NaOH was added to the pellets and heated in a water bath at 100 °C for 30 min to digest the bacterial cell wall. Following cooling to room temperature, the pH was adjusted to seven using 1 M HCl, centrifuged at 4500× *g* for 10 min, and the supernatant containing bioSeNPs was recovered. After centrifugation at 12,000× *g* for 10 min, the supernatant was collected, dialyzed at ambient temperature for 48 h using a dialysis membrane with a molecular weight cut-off of 600 Da, then concentrated by vacuum rotary evaporation and stored at 4 °C. Meanwhile, approximately 100 μL of bioSeNPs solution was coated onto LB plates and incubated at 37 °C for 1–2 days to confirm the absence of bacterial cells.

#### 2.3.2. Stabilization of bioSeNPs with Polysaccharides from the Seeds of *Plantago asiatica* L.

A stock solution of polysaccharides from the seeds of *Plantago asiatica* L. (PLP) at a concentration of 0.1 mg/mL was prepared and filtered through a 0.22 μm filter membrane. Purified bioSeNP solution was diluted to 10 mM with Tris/HCl (pH 7.4). Under magnetic stirring, PLP solution was gradually added to the bioSeNP solution until the mixture’s final concentration was 0.08 mg/mL. The system was subjected to vibrational agitation at ambient temperature for a duration of 10 min. The polysaccharide-decorated biogenic selenium nanoparticle (PLP-bioSeNPs) complex was freeze-dried (ALPHA 1–2; Martin Christ, Osterodeam Harz, Germany) until a constant weight was reached.

### 2.4. Characterization of bioSeNPs and the PLP-bioSeNP Complex

#### 2.4.1. Particle Size and Zeta Potential Analysis

The particle size, polydispersity index (PDI) and zeta potential of bioSeNPs and the PLP-bioSeNP complex were detected using a Zetasizer Nano ZS90 (Malvern Instruments Ltd., Malvern, UK).

#### 2.4.2. Fourier Transform Infrared (FT-IR) Analysis

FT-IR spectra (Nicolet FT-IR 5700; Thermo Electron, Waltham, MA, USA) were used to scan the infrared spectra of bioSeNPs and the PLP-bioSeNP complex in the range 4000–400 cm^−1^ by the potassium bromide pressing method to determine the binding mode of PLP with bioSeNPs. About 5 mg of sample and 100 mg of KBr were mixed to prepare disks.

#### 2.4.3. X-Ray Photoelectron Spectroscopy (XPS) Analysis

The valence state of selenium in the samples was analyzed using XPS (ESCALAB250Xi; Thermo Fisher Scientific, Waltham, MA, USA). The analytical conditions were specified as follows: the vacuum level in the analysis chamber was 5 × 10^−9^ torr. The full-spectrum scanning was conducted with a pass energy of 160 eV, while the narrow-spectrum scanning employed a pass energy of 40 eV. Each sample underwent a single scan. The obtained spectra were calibrated by referencing the C 1s peak at 284.6 eV.

#### 2.4.4. Morphological Analysis

The surface element composition and corresponding proportion of the PLP-bioSeNP complex were observed using scanning electron microscopy-energy dispersive spectroscopy (SEM-EDS, SU8100; Hitachi). The lyophilized B. subtilis ESNS-2 and PLP-bioSeNP complex powder were dispersed on the copper mesh covered with porous carbon film, and observed under a transmission electron microscope (TEM, JEM-2100; Hitachi, Tokyo, Japan).

### 2.5. Determination of In Vitro Antioxidant Activity

The scavenging rates for DPPH and ABTS were calculated using the following formula:

$$\mathrm{Radical}\;\mathrm{scavenging}\;\mathrm{rate}\;(\%)\;=\;\left(1-\frac{Ai-Aj}{Ac}\right)\times 100\%$$

Ai: Absorbance of the sample solution; Aj: Absorbance of the sample blank solution; Ac: Absorbance of the control solution.

#### 2.5.1. DPPH• Free Radical Scavenging Ability

The DPPH• ethanol stocking solution (0.1 mM) was diluted with anhydrous ethanol until its absorbance at 517 nm was less than 0.7. Using vitamin C (Vc) as the positive control, various concentrations (20, 40, 60, 80 and 100 μg·mL^−1^) of bioSeNPs and the PLP-bioSeNP complex were mixed with DPPH• or anhydrous ethanol (blank solution). After incubating in darkness at room temperature for 30 min, the absorbance of mixtures was then measured at 517 nm using a multifunction enzyme labeling instrument (Varioskan Flash; Thermo Fisher Scientific).

#### 2.5.2. ABTS•+ Radical Scavenging Activity

The ABTS•+ stocking solution (7.4 mM) was mixed with an equal volume of potassium persulfate (2.45 mM) and placed in the dark for 12–16 h, then diluted with ultra-pure water until its absorbance at 734 nm was less than 0.7. Using vitamin C (Vc) as the positive control, various concentrations (20, 40, 60, 80 and 100 μg·mL^−1^) of bioSeNPs and the PLP-bioSeNP complex (in terms of selenium concentration) were mixed with ABTS•+ working solution. After incubating in darkness at room temperature for 18 min, absorbance was measured at 734 nm using a multifunction enzyme labeling instrument (Varioskan Flash; Thermo Fisher Scientific).

### 2.6. Amelioration of Oxidative Stress Damage in D-Galactose-Induced Aging Mice

#### 2.6.1. Animal Experiment Design

Thirty eight-week-old SPF male Kunming mice (28–32 g) were purchased from the Tongxiang Branch of Zhejiang Viton Lihua Laboratory Animal Technology Co., Ltd. (No. SCXK(Zhe)2024-0001) (Tongxiang, China). The experiment was approved by the Ethics Committee for Animal Experiments of Nanchang University, China (No. NCULAE-20250623001, date of approval: 23 June 2025) and carried out in accordance with the National Research Council’s Guide for the Care and Use of Laboratory Animals. Mice were kept under standard conditions (room temperature 25 ± 2 °C, relative humidity 50 ± 10%, light–dark cycle for 12 h) for 7 d, and then randomly divided into five groups (six mice/group): the normal control group (Control), D-galactose group (Model), positive control group (vitamin E, VE), bioSeNP group (bioSeNPs) and the PLP-bioSeNP complex group (PLP-bioSeNPs). Except for those in the Control group, all of the mice were subcutaneously injected with 150 mg/kg·bw (body weight) D-galactose at the back of the neck for 12 weeks and gavaged with 0.01 mL/kg·bw of normal saline, 150 mg/kg·bw of vitamin E, 0.5 mg/kg·bw of bioSeNPs (calculated based on selenium) or 0.5 mg/kg·bw of the PLP-bioSeNP complex (calculated based on selenium) at different times daily. The body weights of the mice were measured weekly throughout the experimental period. After the behavioral test, blood was collected by the eyeball blood collection method, and then the mice were slaughtered and the liver and brain samples were rapidly removed and weighed.

#### 2.6.2. Behavioral Test

To minimize stress responses of mice to the experimental environment and apparatus, a habituation protocol was implemented one week prior to behavioral testing. Mice received a gentle manual massage of the dorsal and cervical regions for one minute and were subsequently allowed to freely explore the testing apparatus for five minutes at a fixed time (14:00–15:00) every day.

On the terminal day of the experiment, open-field testing was conducted following intragastric administration. The mice were placed in a 45 × 45 × 15 cm^3^ space and allowed to explore freely for a period of 6 min. Locomotor activity was evaluated based on the total distance they moved and the duration of their exploration. The field was divided into 4 × 4 grid zones using behavioral analysis software, and the mice’s anxiety-like behavior was assessed based on quantifying the time spent in the peripheral zones of the arena.

Short-term spatial working memory was assessed utilizing a Y-maze spontaneous alternation test, conducted two hours after the completion of open-field testing. The Y-maze apparatus comprised three symmetrically arranged arms (120° angular separation, 50 × 32 × 16 cm^3^). Each mouse was placed at the central platform of the Y maze and granted a 5 min exploration period. A complete spontaneous alternation behavior was defined as sequential entries into three distinct arms without repetition. Spontaneous alternation rate (%) = number of spontaneous alternations × 100%/(total number of arm entries − 2).

#### 2.6.3. Histopathological Observation

Liver or brain tissues were fixed in 4% paraformaldehyde solution for 24 h. Subsequently, the tissues were dehydrated through a graded ethanol series, cleared in xylene, embedded in paraffin, and sectioned at 5 µm thickness. After hematoxylin and eosin (HE) staining, the sections were dehydrated, cleared, sealed and examined using an Aperio LV1 real-time digital pathology scanning system (Leica Biosystems, Wetzlar, Germany).

#### 2.6.4. Determination of LPS Concentration

After blood collection, the blood samples were incubated at room temperature for 1 h and subsequently centrifuged at 2000 rpm for 15 min. The resultant supernatant was harvested, and the serum LPS concentration was quantified.

#### 2.6.5. Determination of Antioxidant Parameters and Inflammatory Cytokines

Liver or brain tissues were homogenized in PBS at a tissue-to-buffer ratio of 1:10 (mg:μL), followed by centrifugation at 12,000 rpm for 10 min. The resulting supernatant was collected for analysis of MDA content, GSH, and SOD activities. ELISA kits were used to measure inflammatory cytokines, including TNF-α, IL-6, and IL-1β.

### 2.7. Statistical Analysis

The data were presented as mean ± standard deviation (S.D.) and subjected to statistical analyses using GraphPad Prism 10.6 and SPSS 26 software. Univariate analysis of variance (ANOVA) was performed, followed by Tukey’s or Dunnett’s post hoc tests for multiple comparisons.

## 3. Results

### 3.1. Isolation of Bacillus subtilis

**Figure 1:**
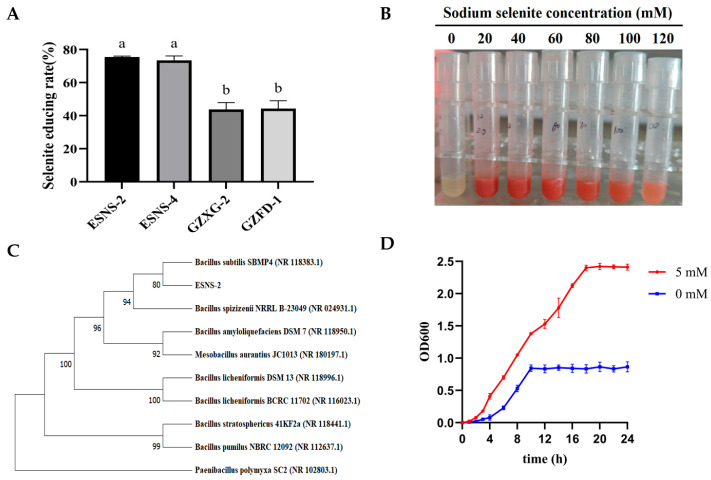
(**A**) Selenite reduction rates of four *Bacillus subtilis* strains over a 48 h incubation period; (**B**) growth assessment of strain ESNS-2 following 24 h cultivation in media containing varying concentrations (mM) of sodium selenite; (**C**) phylogenetic tree constructed from 16S rRNA gene sequences of strain ESNS-2 using the neighbor-joining method; (**D**) comparative growth curves of strain *Bacillus subtilis* ESNS-2 in medium supplemented with or without 5 mM sodium selenite. Data were expressed as mean ± S.D. (*n* = 3). Distinct letters denoted statistically significant differences between experimental groups (*p* < 0.05).

Eight bacterial strains were isolated from soil specimens collected from selenium-rich regions and subsequently subjected to screening for selenite reduction capability. Four of these strains demonstrated a pronounced ability to reduce sodium selenite and were designated as GZXG-2, GZFD-1, ESNS-2 and ESNS-4, respectively. Among these, strain ESNS-2 achieved a reduction of 5 mM sodium selenite by 75.4 ± 0.6% within a 24 h period (Figure 1A). Notably, following 24 h of exposure to a 120 mM sodium selenite solution, strain ESNS-2 retained viability, indicating a pronounced tolerance to elevated selenite concentrations (Figure 1B).

Strain ESNS-2 exhibited distinct morphological characteristics on LB medium, forming circular colonies with irregular margins that were opaque, grayish-white and had a rough surface. Physiological and biochemical analyses indicated that the biochemical profile of strain ESNS-2 aligns consistently with those typical of Bacillus species (Table 1). Furthermore, the amplified 16S rRNA gene fragment was subjected to a BLASTN search against the EzBioCloud database (https://www.ezbiocloud.net/). The strain exhibited high sequence homology with *Bacillus subtilis* SBMP4, thereby confirming its classification as *B. subtilis*, and was designated as *Bacillus subtilis* ESNS-2 (Figure 1C). We further investigated the growth of *Bacillus subtilis* ESNS-2 in the medium supplemented with 5 mM Na_2_SeO_3_. As shown in Figure 1D, in the absence of sodium selenite, the strain exhibited a logarithmic phase from 0 to 4 h, followed by an exponential growth phase from 4 to 10 h, and subsequently entered the stationary phase after 10 h. However, 5 mM of sodium selenite significantly delayed the logarithmic phase of the strain’s growth, with the stationary phase not being reached until 18 h.

### 3.2. Stabilization of bioSeNPs with PLP

**Figure 2:**
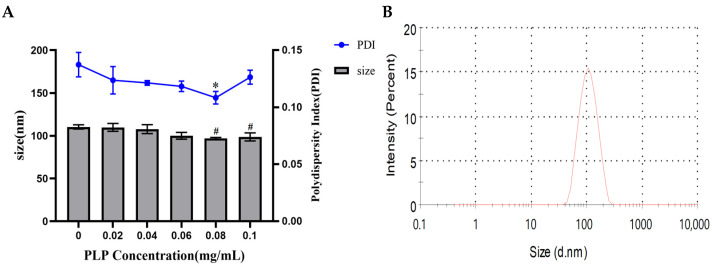
(**A**) Effects of varying PLP concentrations (mg/mL) on the particle size and polydispersity index (PDI) of bioSeNPs. (**B**) Particle size distribution profile of the PLP-bioSeNP complex. Data were presented as mean ± S.D. (*n* = 3). Statistical significance for PDI and particle size was denoted as * *p* < 0.05 and ^#^ *p* < 0.05 in comparison to the Control group, respectively.

Particle size refers to the size of nanoparticles, which is an important indicator of the activity of SeNPs. The PDI measures the range of the particle size distribution in a colloidal solution, which is inversely proportional to the uniformity of the colloidal solution. A higher PDI value denotes a broader and less uniform particle size distribution in the solution. Typically, a PDI value below 0.3 indicates excellent monodispersity and a narrow distribution of the nanoparticles, while a PDI value lower than 0.1 suggests that the colloidal solution approaches complete uniformity, reflecting a highly homogeneous particle population [34]. Zeta potential refers to the electrical charge on the particle surface, and an absolute zeta potential value exceeding 20 mV indicates that the system exhibits relative stability [35]. As depicted in Figure 2A, the purified bioSeNPs exhibited an average particle size of 110.3 ± 2.4 nm, a PDI of 0.137 ± 0.011, and a zeta potential of −14.5 ± 0.5 mA. These parameters indicated a moderate degree of colloidal dispersibility. However, the relatively low magnitude of the surface charge implied that the long-term stability of the bioSeNPs may potentially be compromised. The incorporation of PLP resulted in a reduction in the PDI of bioSeNPs. As the PLP concentration increased, the PDI of the PLP-bioSeNP complex displayed an initial decrease followed by a subsequent increase. At a PLP concentration of 0.08 mg/mL, the particle size of the PLP-bioSeNP complex was reduced to 96.9 ± 1.1 nm, with a PDI of 0.108 ± 0.003, demonstrating enhanced colloidal stability compared to the system before PLP introduction (*p* < 0.05). The zeta potential of −19.7 ± 0.4 mA further corroborated that the prepared PLP-bioSeNP complex exhibits high homogeneity and excellent colloidal stability.

### 3.3. Characterization of the PLP-bioSeNPs Complex

#### 3.3.1. Morphology and Elemental Distribution of the PLP-bioSeNP Complex

**Figure 3:**
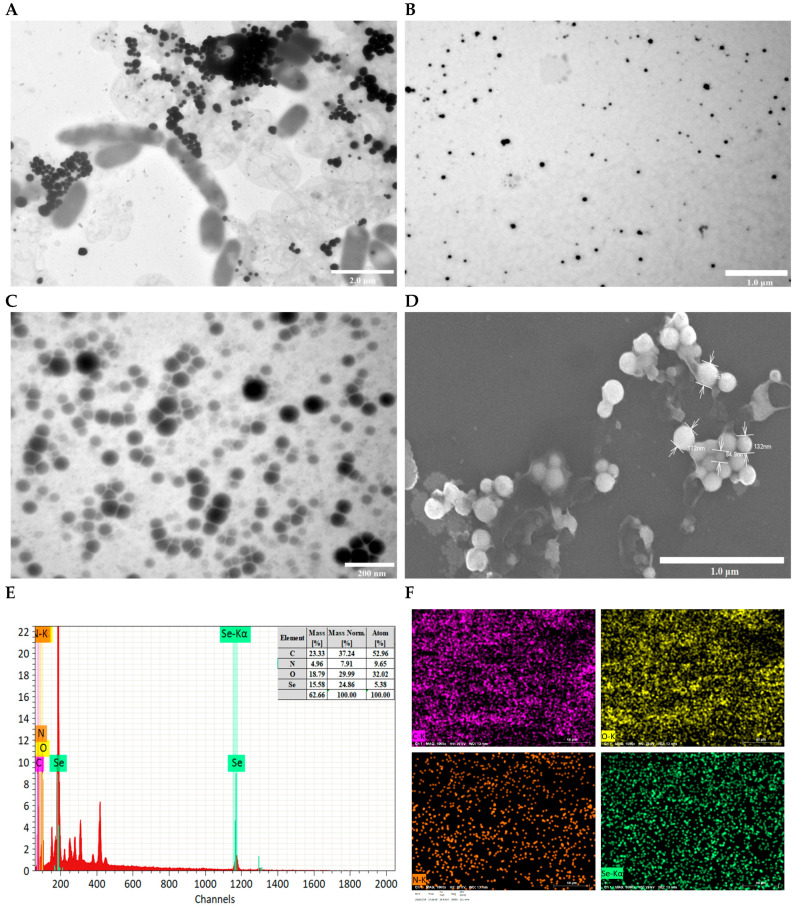
(**A**) TEM image of bioSeNPs synthesized by strain Bacillus subtilis ESNS-2; (**B**,**C**) TEM images depicting the PLP-bioSeNP complex; (**D**) SEM image of the PLP-bioSeNP complex; (**E**) EDS full-range spectrum and corresponding quantitative compositional analysis of the PLP-bioSeNP complex; (**F**) elemental mapping for C, O, N and Se.

The TEM imaging of *B. subtilis* ESNS-2 cells after 48 h of incubation with sodium selenite demonstrated the existence of black spherical particles localized both on the periphery of and within the extracellular space surrounding bacterial cells (Figure 3A). These particles exhibited diameters ranging from 50 to 400 nm, which is consistent with the size range of SeNPs synthesized by *Lysinibacillus bacilli* (200–500 nm in diameter) or *Pediococcus acidilactici* (50–400 nm in diameter) [36,37]. After introducing polysaccharides, the PLP-SeNP complex demonstrated a homogeneous distribution without any indications of agglomeration. Upon magnification, these particles presented as regular spheres with distinct contours and a uniform size, primarily ranging from 30 to 80 nm (Figure 3B,C). This observation corroborates the DLS results, indicating that the sample exhibits good overall dispersion and that the polysaccharide modification effectively suppresses the excessive agglomeration of the SeNPs. The SEM image showed that nanoparticles exhibited a regular spherical shape with smooth surfaces (Figure 3D). In addition, a translucent matrix was visible on the particle surfaces and gaps, indicating the successful coating of PLP on the SeNP surfaces. This result also underscored the critical role of PLP in promoting the dispersion and stabilization of SeNPs. The EDS mapping analysis confirmed the presence of selenium, indicating an oxidation state of elemental selenium (Se^0^), as evidenced by the characteristic Se Kα and Se Lα peaks at 11.21 keV and 1.38 keV, respectively [38]. Moreover, EDS element mapping images show the uniform distribution of carbon (C), nitrogen (N), oxygen (O) and selenium (Se) in the PLP-bioSeNP complex particles, with respective mass percentages of 37.24%, 7.91%, 29.99%, and 24.86% (Figure 3E). These results demonstrated the uniform and effective binding between bioSeNPs and PLP, while the higher combined content of C and O (approximately 67.23%) aligns with the chemical composition of PLP, and the selenium content unequivocally substantiates the presence of the bioSeNP core.

#### 3.3.2. Stabilization Mechanism of the PLP-bioSeNP Complex

**Figure 4:**
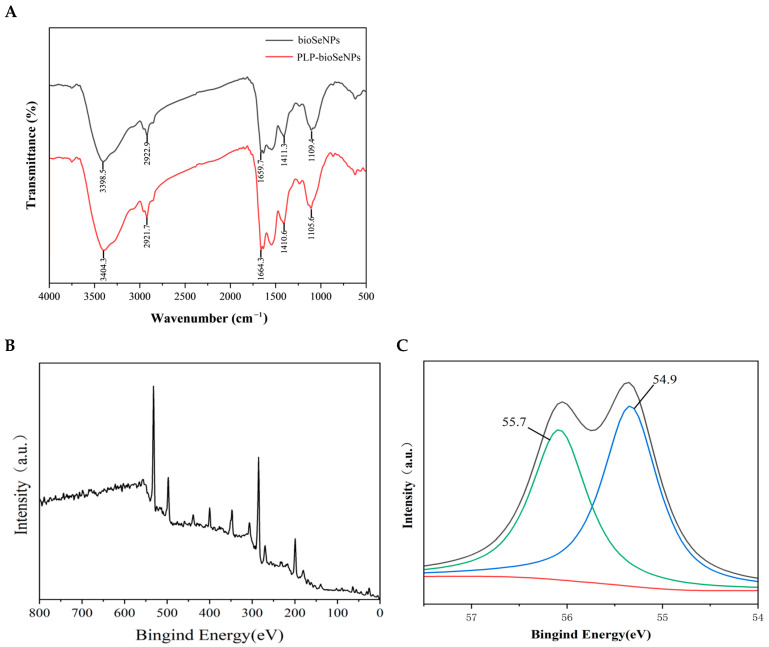
(**A**) FT-IR spectra depicting the characteristic vibrational signatures of bioSeNPs and the PLP-bioSeNP complex; (**B**) XPS full-scan profile and (**C**) Se 3d split-peak fitting profile of the PLP-bioSeNP complex.

As depicted in Figure 4A, the characteristic C-H and C-O absorption peaks of bioSeNPs and the PLP-bioSeNP complex overlapped at 2921.7 cm^−1^ and 1105.6 cm^−1^, respectively, suggesting that PLP is bound to bioSeNPs. A markedly enhanced and red-shifted O-H absorption peak at 3404.3 cm^−1^ for the PLP-bioSeNP complex relative to bioSeNPs further indicates a strong hydrogen bonding interaction (O-H⋯Se) between the hydroxyl groups of PLP and bioSeNPs [39]. The absorption peak at 1659.7 cm^−1^ corresponds to the amide I band (C=O stretching vibration) of residual bacterial proteins derived from the biosynthetic process [40].

Full-scan XPS mapping revealed a characteristic peak corresponding to Se within the low-binding energy region, while characteristic peaks for C, N, and O elements were detected at binding energies of 285 eV, 400 eV, and 532 eV, respectively [41]. These findings indicate the presence of bioorganic components, such as polysaccharides and proteins, which is consistent with the results obtained from EDS and FT-IR analyses. The Se 3d peak fitting map elucidated a characteristic peak corresponding to elemental selenium (Se^0^) at 55.1 eV for the Se 3d5/2 orbital. Further analysis indicated that elemental selenium (Se^0^) was responsible for the electron binding energy peaks observed for Se 3d5/2 and Se 3d3/2, which were measured at 55.4 eV and 56.3 eV, respectively, as confirmed by comparison with the NIST X-ray Photoelectron Spectroscopy Database-Standard Reference Database-Standard Reference Database 20 (Version 4.1) [42]. The double peak at 54.9 eV and 55.7 eV corresponds to amorphous or surface-coordinated Se^0^ and crystalline Se^0^, respectively (Figure 4C). The absence of a detected electron binding energy peak for Se 3d5/2 at 59.1 eV suggested that no characteristic peaks of selenite (Se^4+^) or selenate (Se^6+^) were present. These findings demonstrate that the reaction within the solution system proceeded to completion, resulting in the full reduction in highly toxic sodium selenite to low-toxicity elemental selenium (Se^0^). Based on the above analytical results, the stabilization mechanism of PLP-bioSeNPs has been elucidated as follows: subsequent to the biosynthesis of SeNPs by Bacillus subtilis ESNS-2, the bioSeNPs, encapsulated within a biological matrix, were isolated and further passivated via non-covalent interactions, predominantly hydrogen bonding, with PLP. This process effectively inhibited the aggregation of bioSeNPs, thereby yielding a well-dispersed and stable PLP-bioSeNP complex.

### 3.4. Stability Analysis of the PLP-bioSeNP Complex

**Figure 5:**
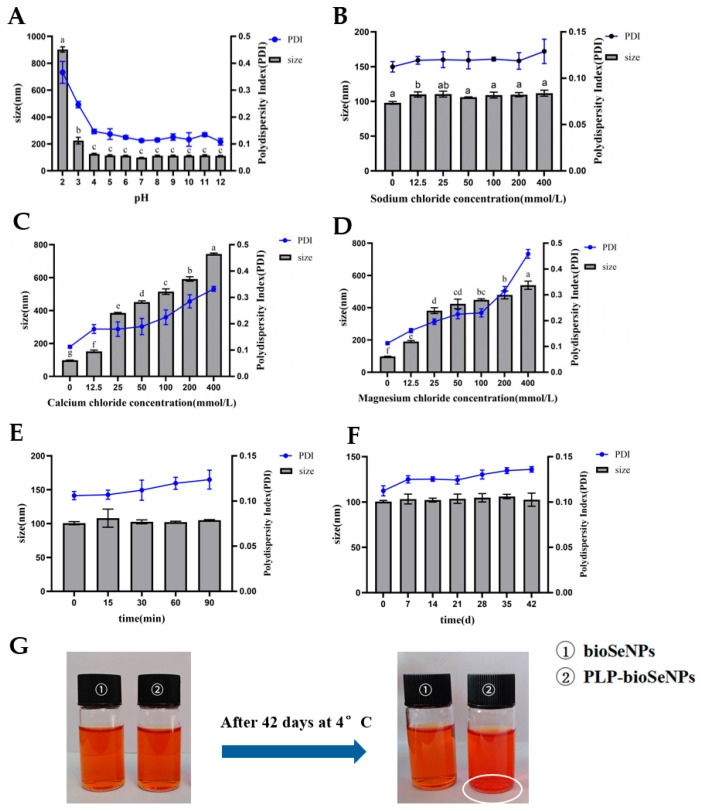
Influence of varying pH values (**A**), NaCl concentration (**B**), CaCl_2_ concentration (**C**), and MgCl_2_ concentration (**D**) on alterations in particle size and PDI of the PLP-bioSeNP complex; assessment of storage stability of PLP-bioSeNPs at 60 °C (**E**) or 4 °C (**F**); photographs of bioSeNPs and the PLP-bioSeNP complex stored at 4 °C after 0 and 42 days (**G**). Data were expressed as mean ± S.D. (*n* = 3). Distinct letters denote statistically significant differences between experimental groups (*p* < 0.05).

Given that stability serves as a critical determinant of the bioactivity and applications of nanomaterials [43], we assessed the stability of the PLP-bioSeNP complex by monitoring alterations in the PDI and particle size. As shown in Figure 5A, the PLP-bioSeNP complex exhibited poor stability under strong acidic conditions (pH 2~3), with a significantly enlarged particle size and pronounced aggregation. However, both the particle sizes and the PDI of the PLP-bioSeNP complex remained at low levels, with the system maintaining stability as the pH increased from four to 12. These results demonstrated that the PLP coating confers effective protection to bioSeNP, and the PLP-bioSeNP complex exhibits robust stability under both weakly acidic and alkaline conditions. The PLP-bioSeNP complex demonstrated superior stability in sodium chloride solutions at concentrations ranging from 0 to 400 mM (Figure 5B). In contrast, both particle size and PDI of the PLP-bioSeNP complex underwent significant alterations and exhibited a pronounced aggregation upon reaching a calcium chloride or magnesium chloride concentration of 25 mM (Figure 5C,D). As depicted in Figure 5E, both the particle sizes and PDI of the PLP-bioSeNP complex had no significant increase at a higher temperature (60 °C) for 90 min. Unlike bioSeNPs, throughout a 42-day storage period, the PLP-bioSeNP complex solution exhibited notable stability, with no significant precipitation observed (Figure 5F,G). In summary, the PLP-decorated bioSeNPs demonstrated remarkable storage stability under diverse environmental conditions and over an extended duration. Furthermore, they retained stability during mild thermal processes, such as pasteurization.

### 3.5. In Vitro Antioxidant Activity

**Figure 6:**
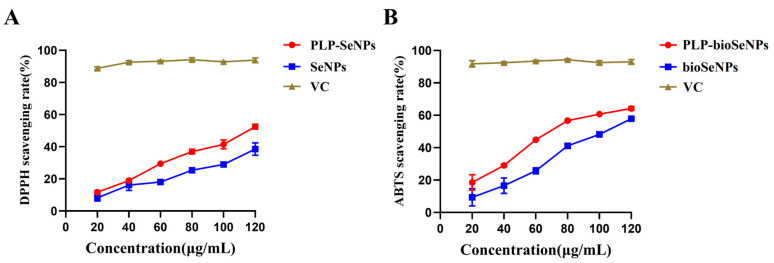
Antioxidant activities of bioSeNPs and the PLP-bioSeNP complex by measuring DPPH• radical (**A**) and ABTS^+^• radical (**B**) scavenging capacity. VC was a positive control. Data were expressed as mean ± S.D. (*n* = 3).

As depicted in Figure 6, both the PLP-bioSeNP complex and bioSeNPs demonstrated a pronounced dose-dependent scavenging efficacy against DPPH• and ABTS^+^• radicals, with radical scavenging capacity increasing significantly as sample concentration elevated. Notably, at equivalent concentrations, the radical scavenging capacity of PLP-bioSeNPs was consistently slightly higher than that of bioSeNPs. This may be attributed to the inherent radical scavenging ability of PLP [28], which may synergistically enhance the antioxidant effect with bioSeNPs. Furthermore, the comparatively smaller particle size, enhanced specific surface area, and heightened chemical reactivity of PLP-bioSeNPs relative to bioSeNPs may also constitute a contributing factor.

### 3.6. In Vivo Anti-Aging Activity

#### 3.6.1. Effects of bioSeNPs and the PLP-bioSeNP Complex on Body Weight Changes and Organ Indices in Aging Mice

As a well-established animal model for aging research, the administration of D-galactose has been demonstrated to induce memory and cognitive impairments in mice, accompanied by aging-associated deficits [44]. At the end of the experiment, mice in the Control group maintained optimal physiological condition, exhibiting prompt behavioral responses and smooth, glossy fur throughout the experimental period. While mice in the Model group showed skin laxity, rough and erect fur, and diminished responsiveness to external stimuli following prolonged D-galactose administration (approximately 30 days). Compared to the Model group, aging mice in the bioSeNP, PLP-bioSeNP, and VE groups demonstrated improved behavioral performance and restored fur luster.

**Figure 7:**
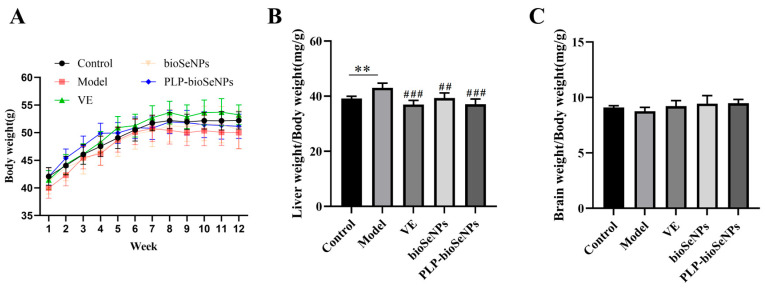
(**A**) Body weight change curve of mice (*n* = 8); liver index (*n* = 6, (**B**)) and brain index (*n* = 6, (**C**)) were measured and calculated at the end of the 12-week experimental period, following the behavioral tests. Data were presented as mean ± S.D. ** *p* < 0.05 compared to the Control group; ^###^ *p* < 0.001; ^##^ *p* < 0.01 compared to the Model group.

As shown in Figure 7A, the body weights of all groups exhibited similar trends, with gradual and steady increases that ceased upon reaching adulthood, showing no significant differences among the groups. Compared to the Control group, the liver organ index in the Model group exhibited a statistically significant increase, which is consistent with previous findings that D-galactose-induced oxidative stress caused substantial hepatic damage, followed by inflammatory responses and ultimate hepatic edema [45]. However, dietary bioSeNPs, PLP-bioSeNPs, or VE supplementation significantly alleviated the liver-to-body mass ratio as compared to the Model group mice, whereas no significant difference in the brain-to-body mass ratio was observed between groups (Figure 7B).

#### 3.6.2. Effects of PLP-bioSeNPs and bioSeNPs on Behavioral Characteristics in Aging Mice

**Figure 8:**
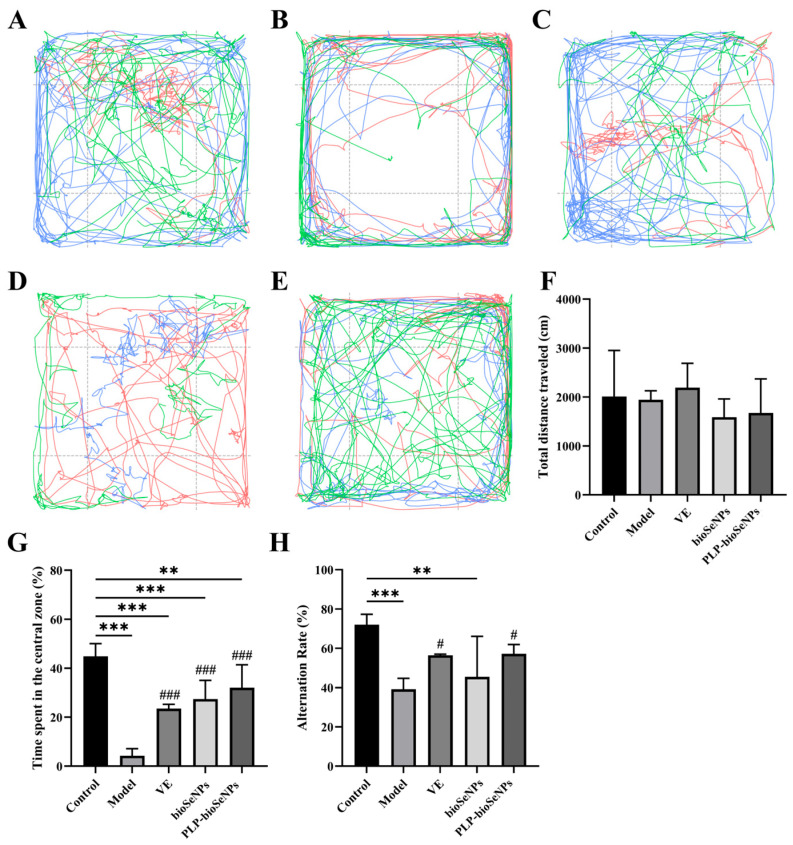
Movement trajectories of mice in the open-field test (three representative individuals were randomly selected from each group): (**A**) Control group; (**B**) Model group; (**C**) VE group; (**D**) bioSeNP group; (**E**) PLP-bioSeNP group; (**F**) total moving distance of mice; (**G**) percentage of residence time in the central area; (**H**) spontaneous alternation rate in the Y-maze test. Data were presented as mean ± S.D. (*n* = 6). ** *p* < 0.01; *** *p* < 0.001 compared to the Control group; ^#^ *p* < 0.05; ^###^ *p* < 0.001 compared to the Model group.

The spontaneous activity and anxiety-like behavior of mice were evaluated using open-field experiments. Under typical conditions, animals engage in active exploration of novel environments, whereas those with cognitive impairments exhibit reduced locomotor activity in new environments [46]. Representative movement trajectories of mice in each group are shown in Figure 8A–E. No significant differences in total movement distance were detected among the groups, suggesting that neither D-galactose or various intervention treatment affects the spontaneous motor ability of the mice, thereby precluding potential interference from motor deficits on subsequent behavioral measures (Figure 8F). Compared to the Control group, mice in the Model group exhibited a significant reduction in the proportion of time spent in the central area, accompanied by marked wall-seeking behavior, indicating the successful induction of anxiety-like emotions. In contrast, either VE, PLP-bioSeNPs, or bioSeNPs treatment significantly increased the proportion of time spent in the central area (*p* < 0.05), suggesting an effective alleviation of anxiety-like behaviors and an enhancement of exploratory activity levels in the Model mice. Notably, the PLP-bioSeNP group showed pronounced improvements, indicating that PLP-bioSeNPs may have superior efficacy in mitigating oxidative stress-related mood disorders compared with bioSeNPs (Figure 8G).

Regarding spatial working memory capacity, normal mice in the Control group exhibited a high rate of spontaneous alternation, indicative of robust spatial working memory capacity (Figure 8H). Compared to the Control group, the spontaneous alternation rate in mice of the Model group was significantly lower (*p* < 0.05), indicating that D-galactose-induced oxidative stress impaired neuronal function in cognition-related brain regions such as the hippocampus, leading to the deterioration of spatial working memory. Compared with the Model group, a 12-week intervention with either VE, PLP-bioSeNPs, or bioSeNPs demonstrated a trend toward recovery in spontaneous alternation rates in mice, although none of these differences attained statistical significance (*p* > 0.05).

#### 3.6.3. Effects of PLP-bioSeNPs and bioSeNPs on Antioxidant Level in the Brain and Liver from Aging Mice

**Figure 9:**
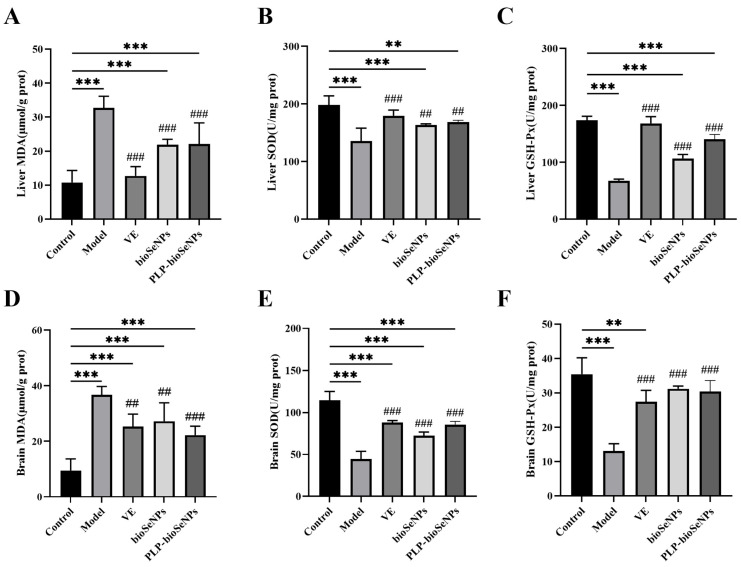
Effects of bioSeNPs and the PLP-bioSeNP complex on D-galactose-induced accumlation of lipid peroxidation (MDA) and the downregulation of antioxidant enzyme (SOD and GSH-Px) activity in liver and brain tissues from aging mice. (**A**) Liver MDA; (**B**) liver SOD; (**C**) liver GSH-Px; (**D**) brain MDA; (**E**) brain SOD; (**F**) brain GSH-Px. Data were presented as mean ± S.D. (*n* = 6). ** *p* < 0.01; *** *p* < 0.001 compared to the Control group; ^##^ *p* < 0.01, ^###^ *p* < 0.001 compared to the Model group.

Growing evidence indicates that oxidative stress is a pivotal determinant of the aging process and the pathogenesis of various age-related diseases [47,48]. Oxidative damage occurs when generated free radicals exceed the capacity of endogenous antioxidant defenses. However, organisms possess multiple antioxidants and antioxidant defense systems, such as SOD, GSH-Px, CAT, vitamin C, and vitamin E, collectively to prevent oxidative damage and mitigate oxidative stress [49]. Therefore, the level of MDA, as an indicator of lipid peroxidation, along with the activities of SOD and GSH-Px in the brain and liver tissues of aging mice, was investigated to assess the systemic oxidative stress status. As illustrated in Figure 9, compared to the Control group, mice in the Model group exhibited significantly elevated MDA levels alongside markedly diminished activities of SOD and GSH-Px in both liver and brain tissues. These results indicated that D-galactose administration disrupted the balance between oxidative and antioxidative processes in mice, thereby inducing substantial lipid peroxidative damage within the hepatic and brain tissues. In comparison with the Model group, all intervention groups exhibited a significant reduction in MDA levels and a marked enhancement in the activities of SOD and GSH-Px, with consistent trends observed in both hepatic and cerebral tissues. Among the interventions, VE administration notably restored various oxidative stress markers in the liver to levels comparable to those in normal mice. More significantly, the values of liver GSH-Px and brain SOD in the PLP-bioSeNP group were higher than those in the bioSeNP group. These findings demonstrated that both bioSeNPs and the PLP-bioSeNP complex effectively ameliorated oxidative damage in the liver and brain tissues of aging mice through the enhancement of antioxidant enzyme activities.

#### 3.6.4. Effects of PLP-bioSeNPs and bioSeNPs on Inflammation Level in the Brain and Liver from Aging Mice

**Figure 10:**
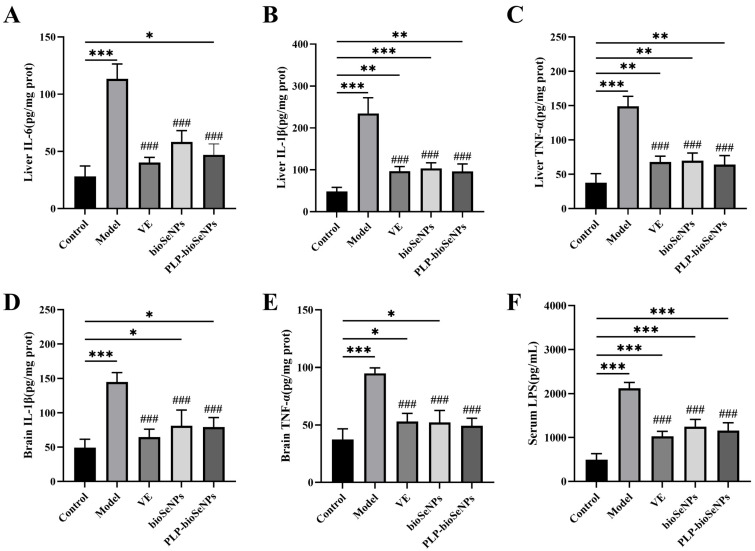
Effects of bioSeNPs and the PLP-bioSeNP complex on pro-inflammatory factors in hepatic and cerebral tissues, as well as serum LPS levels, in D-galactose-induced aging mice. (**A**) Liver IL-6; (**B**) liver IL-1β; (**C**) liver TNF-α; (**D**) brain IL-1β; (**E**) brain TNF-α; (**F**) serum LPS. Data were presented as mean ± S.D. (*n* = 6). * *p* < 0.05; ** *p* < 0.01; *** *p* < 0.001 compared to the Control group; ^###^ *p* < 0.001 compared to the Model group.

Previous studies have confirmed that D-galactose-induced oxidative stress promoted the overexpression of pro-inflammatory factors and damaged the intestinal mucosal barrier, thereby facilitating LPS to enter the bloodstream and eliciting a subsequent systemic inflammatory response. In turn, inflammation accelerated the accumulation of ROS, leading to the formation of a vicious circle and an oxidative stress–inflammatory axis [50,51]. Consequently, serum LPS levels, which constitute a critical biomarker for evaluating intestinal barrier integrity and systemic inflammatory state, along with pro-inflammatory factors in hepatic and cerebral tissues, were quantified. As illustrated in Figure 10F, serum LPS levels were markedly elevated in the Model group relative to the Control group. In contrast, the administration of either PLP-bioSeNPs or bioSeNPs significantly attenuated serum LPS concentrations, exhibiting an effect comparable to that of the positive control VE (vitamin E, 150 mg/kg·bw) (*p* > 0.05). Owing to the factor that oxidative stress can activate inflammatory signaling pathways, leading to the massive release of pro-inflammatory factors such as IL-6, IL-1β, and TNF-α, thereby exacerbating tissue damage, the levels of IL-6, IL-1β, and TNF-α in liver and brain tissues were also quantified [52]. Compared to the Control group, the levels of IL-6, IL-1β, and TNF-α in the livers of the Model group mice, as well as those of IL-1β and TNF-α in brain tissue, were significantly elevated, indicating that D-galactose-induced oxidative stress triggered pronounced inflammatory responses in both liver and brain tissues. A 12-week intervention of either PLP-bioSeNPs or bioSeNPs demonstrated pronounced antioxidant effects by remarkably reducing the pro-inflammatory factors, which was comparable to that of the positive control VE. The reduction in hepatic IL-6 and cerebral TNF-α levels was more pronounced in the PLP-bioSeNP group relative to the bioSeNP group, suggesting enhanced bioactivity of the PLP-bioSeNP complex.

#### 3.6.5. Protects of PLP-bioSeNPs and bioSeNPs on Hippocampus and Liver Harm from Aging Mice

**Figure 11:**
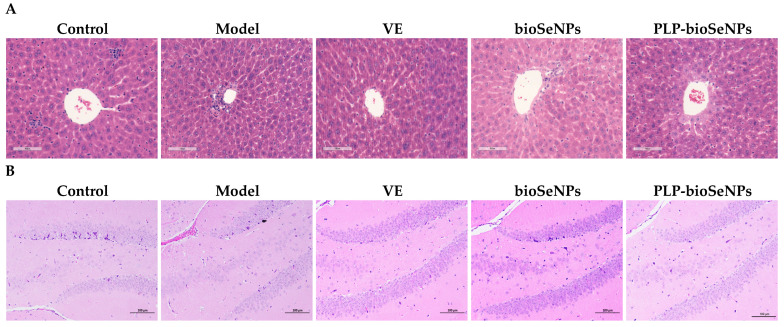
Influence of bioSeNPs and the PLP-bioSeNP complex on histopathological alterations in hippocampal (**A**) and hepatic (**B**) tissues from D-galactose-induced aging mice (H&E staining).

Studies have found that the number and density of neurons in the hippocampus undergo differential alterations with aging, eventually leading to cerebral atrophy and cognitive decline, particularly in memory functions [53]. As depicted in Figure 11, pyramidal neurons in the hippocampal region of mice in the Model group exhibited a disorganized spatial arrangement, appearing loosely distributed and disordered, with notably widened intercellular spaces. This pattern exhibits a marked contrast to the characteristic band-like alignment observed in the Control group, suggesting that D-galactose induced mild neuronal damage in mice. Intervention with VE (vitamin E, positive control), PLP-bioSeNPs, or bioSeNPs for 12 weeks resulted in an amelioration of hippocampal pathological conditions in aging mice. These findings suggested that both PLP-bioSeNPs and bioSeNPs may mitigate early brain injury and ameliorate age-related neurodegenerative pathologies.

D-galactose had been reported to induce hepatic injury and dysfunction, consequently resulting in comprehensive morphological alterations [54]. The H&E staining of mouse liver showed that in contrast to the Control group, hepatocytes in the Model group exhibited a disorganized arrangement, swelling with loose cytoplasm, and local inflammatory cell infiltration around the central veins, indicating that D-galactose successfully induced oxidative stress in mice, leading to liver tissue damage. Following a 12-week treatment regimen with VE, PLP-bioSeNPs, or bioSeNPs, varying degrees of mitigation were observed. The VE group exhibited reduced inflammatory infiltration and a more regular arrangement of hepatocytes, whereas the bioSeNP group exhibited weaker alleviation of pathological damage, with persistent foci of inflammatory cell accumulation remaining discernible. The PLP-bioSeNP group maintained an intact hepatic tissue structure, with hepatocyte morphology approximating that of the normal Control group, and no significant inflammatory infiltration was detected. The findings demonstrated that the PLP-bioSeNP complex significantly alleviated D-galactose-induced hepatic inflammation and structural damage, exhibiting a protective efficacy superior to that of individual bioSeNPs.

## 4. Discussion

In this study, a highly selenite-tolerant strain, *Bacillus subtilis* ESNS-2, was isolated from selenium-rich soil in Enshi, Hubei Province. This strain demonstrated a remarkable capacity to reduce 75.4% of 5 mM sodium selenite within 24 h and could tolerate up to 120 mM, significantly outperforming previously reported strains such as *Bacillus subtilis* J-2 (73.83% reduction after optimization) and *Bacillus subtilis* T5 (approximately 73% reduction of 5 mM selenite in 36 h) [55,56]. The superior selenite-reducing capability of ESNS-2 may be attributed to its long-term adaptation to a high-selenium environment, which could have driven the evolution of efficient enzymatic reduction systems [57]. Growth curve analysis confirmed that selenite exerts oxidative toxicity and interferes with key metabolic enzymes, consistent with previous reports that selenite can inhibit bacterial growth by inducing oxidative stress and disrupting cellular redox homeostasis [58].

To address the limited long-term stability of purified bioSeNPs, polysaccharides from *Plantago asiatica* L. (PLP) were employed as a natural stabilizer. The concentration-dependent effect of PLP on PDI (initial decrease followed by increase) can be explained by the fact that excessive PLP molecules may self-entangle and aggregate, indirectly disrupting the overall system stability. This indicates that stabilization is mediated by hydrogen bonding between PLP hydroxyl groups and SeNPs, combined with steric hindrance, rather than by concentration alone [59]. Characterization studies confirmed that PLP binds to bioSeNPs via non-covalent hydrogen bonding without altering the zero-valent state of selenium, consistent with previous findings for polysaccharide–selenium nanoconjugates [60,61]. The PLP-bioSeNP complex exhibited robust stability under a range of environmental conditions, including weakly acidic to alkaline pH, mild thermal processing, and prolonged storage, though it was sensitive to strong acid and divalent cations.

The in vitro antioxidant assays demonstrated that PLP-bioSeNPs exhibited superior scavenging activity against DPPH• and ABTS^+^• radicals compared to unmodified bioSeNPs, which can be attributed to the inherent antioxidant capacity of PLP [32] and the enhanced specific surface area resulting from reduced particle size. This finding aligns with the established principle that the antioxidant activity of SeNPs is inversely correlated with particle size [62].

The in vivo anti-aging effects were evaluated using a D-galactose-induced aging mouse model. The observed elevation in MDA levels, suppression of antioxidant enzyme activities, and histopathological damage in the Model group confirmed successful model establishment [63]. Oxidative stress is intimately linked to inflammation. Excessive ROS can activate the NF-κB signaling pathway, promoting the release of pro-inflammatory cytokines such as IL-6, IL-1β, and TNF-α, which in turn exacerbate oxidative damage, forming a vicious “oxidative stress–inflammation” cycle [64]. In the present study, intervention with either PLP-bioSeNPs or bioSeNPs effectively reversed pathological changes, reduced pro-inflammatory cytokine levels and serum LPS, demonstrating their capacities to scavenge excess ROS, break the “oxidative stress–inflammation” cycle and restore the endogenous antioxidant defense system. Notably, although PLP-bioSeNPs showed statistically significant advantages over bioSeNPs in certain parameters (e.g., liver GSH-Px, brain SOD), the overall anti-aging effect did not reach statistical significance between the two groups. This may be attributable to residual bioactive macromolecules from bacterial cells narrowing the difference in intestinal activity.

Compared to chemically synthesized SeNPs stabilized with synthetic polymers, PLP-decorated biologically synthesized SeNPs offer the advantages of green synthesis, enhanced biocompatibility, and improved storage stability. Future studies incorporating molecular investigations, gut microbiota analysis, and long-term toxicity assessments will be valuable for advancing the application of PLP-bioSeNPs in functional foods and animal nutrition.

## 5. Conclusions

In summary, *Bacillus subtilis* ESNS-2, isolated from selenium-rich soil, efficiently reduced sodium selenite to produce spherical bioSeNPs with a size of about 110.3 ± 2.4 nm. After the addition of polysaccharides extracted from the seeds of *P. asiatica* L. (PLP), PLP-bioSeNPs were formed with excellent dispersibility and stability. In vitro, both bioSeNPs and PLP-bioSeNPs exhibited potent, dose-dependent radical scavenging activity against DPPH• and ABTS^+^• radicals, with PLP-bioSeNPs demonstrating superior efficacy. Moreover, both bioSeNPs and PLP-bioSeNPs alleviated D-galactose-induced hepatic and cerebral damage, as well as associated behavioral deficits, through the modulation of oxidative stress balance and suppression of inflammation. These results demonstrate that the production of PLP-bioSeNPs via microbial fermentation coupled with polysaccharide modification possess robust antioxidant and anti-aging properties, presenting a promising strategy for scalable and cost-effective manufacturing, with potential applications in animal nutrition and the food industry.

## Figures and Tables

**Table 1:** Physiological and biochemical characteristics of the strain ESNS-2. + Positive; − Negative.

Characteristics	Results
Anaerobic growth	−
V-P test	+
Citrate utilization	+
Propionate utilization	−
D-xylose utilization	+
L-arabinose utilization	+
D-mannitol utilization	+
Gelatin liquefaction test	+
7% sodium chloride tolerance	+
pH 5.7 tolerance	+
Nitrate reduction test	+
Amylohydrolysis test	+

## Data Availability

The data presented in this study are available on request from the corresponding author.
